# Effect of prostate volume on the peripheral nerve block anesthesia in the prostate biopsy: A strobe-compliant study

**DOI:** 10.1097/MD.0000000000004184

**Published:** 2016-07-18

**Authors:** Yang Luan, Tian-bao Huang, Xiao Gu, Guang-Chen Zhou, Sheng-Ming Lu, Hua-Zhi Tao, Bi-De Liu, Xue-Fei Ding

**Affiliations:** Department of Urology, Clinical Medical School, Yangzhou University, Yangzhou, Jiangsu Province, China.

**Keywords:** biopsy, periprostatic nerve block anesthesia, prostatism

## Abstract

**Objective:**

The objective of this study was to evaluate the anesthetic efficacy of periprostatic nerve block (PNB) in transrectal ultrasound (TRUS)-guided biopsy on different prostate volume.

**Methods:**

A total of 568 patients received prostate biopsy in our hospital from May 2013 to September 2015 and were retrospectively studied. All patients were divided into local anesthesia group (LAG) and nerve block group (NBG). Then each group was subdivided into 4 subgroups (20–40, 40–60, 60–100, and >100 mL groups) according to different prostate volume range. Visual analogue scale (VAS) and visual numeric scale (VNS) were used to assess the patient's pain and quantify their satisfaction. The scores and complications were compared between the groups.

**Results:**

The age and serum prostate-specific antigen (PSA) level before biopsy had no significant differences at intergroup or intragroup level. The VAS scores were significantly lower in the NBG than those in the LAG in terms of prostate volume (1 (1–2) versus 2 (1–3), 2 (1–3) versus 2 (2–4), 2 (2–3) versus 3 (2–5), 4 (3–5) versus 5 (4–7), all *P* < 0.05). Conversely, the VNS scores were higher in the NBG (4 (3–4) versus 3.5 (3–4), 3 (3–4) versus 3 (3–3), 3 (2–4) versus 3 (2–3), 2 (2–2) versus 1 (1–2), all *P* < 0.05). Patients with smaller prostate volume undergoing PNB or local anesthesia experienced significantly lower pain and higher satisfaction scores than those with large prostate. Whether in PNB or local anesthesia group, patients with large prostate volume had more chance to have hematuria, hemospermia, urinary retention than smaller one except infection (*P* < 0.05). Those complications had no significant differences between LAG and NBG (*P* > 0.05).

**Conclusion:**

Compared with local anesthesia, ultrasound-guided PNB has superior analgesic effect and equal safety, but for patients with a large prostate volume, the analgesic effect is inefficient.

## 1 Introduction

Prostate cancer (PCa) is the second leading cause of cancer-related deaths in American men.^[1]^ The incidence of PCa in China is lower than that of the western countries, but with the improvement of living standards, the change of dietary pattern, the increase of population aging and the popularity of PCa screening methods, the incidence increases annually and the trend of younger age appear significantly.^[2]^ Screening for PCa can significantly reduce the related morbidity and increase the duration of lifetime.^[3]^ Therefore, early diagnosis and effective treatment of PCa is of great significance to prolong the survival time and improve the quality of life of patients with PCa.^[4]^ Previous studies found that transperineal template-guided prostate biopsy was the effective way to improve the early detection rate of tumor, compared with the TRUS-guided prostate biopsy.^[5,6]^ Owing to use of template location, puncture needle is always parallel to endorectal ultrasound probe. Full monitoring of the needle road and the depth of the needle can improve the safety of biopsy, which can lead to the possibility of increasing puncture number. As is known, the positive rate is increased by the puncture number within a certain range.^[6,7]^ The puncture, in this way, passes through the skin and subcutaneous layers, which might increase the puncture time. Hence, patients may inevitably suffer from more pain than the transrectal puncture.^[8]^ About 20% of patients rejected prostate biopsy without anesthesia.^[9]^ So effective anesthesia before prostate puncture was a prerequisite.^[10]^ We retrospectively reviewed the clinical data of 568 patients with prostate biopsy in our hospital from May 2013 to September 2015, and assessed the efficacy and safety of PNB, compared with the local anesthesia. Besides, the effect of prostate volume was also analyzed.

## 2 Materials and methods

### 2.1 Clinical data

In this study, 568 patients who underwent prostate biopsy from May 2013 to September 2015 in our hospital were included. Puncture indications: digital rectal examination finds nodules with any prostate-specific antigen (PSA) value; B ultrasound or CT or MRI examination reveals abnormal images with any PSA value; PSA >10 μg/L with any free/total (f/t) PSA and prostate-specific antigen density (PSAD); PSA range from 4 to 10 μg/L with abnormal f/t PSA or PSAD value. All patients were divided into LAG and NBG according to anesthesia methods. Then each group was subdivided into 4 subgroups (20–40, 40–60, 60–100, and >100 mL subgroups) according to different prostate volume range. Finally, 304 patients who underwent PNB and 264 patients who used local anesthesia were included in our study. Before prostate biopsy, the patients of LAG had prostate-coated local anesthesia. Whereas in NBG, patients received additional PNB apart from prostate-coated local anesthesia. All clinical data used in this study were approved by the Ethics Committee of Clinical Medical School of Yangzhou University. Written informed consent was obtained from each patient before prostate biopsy.

### 2.2 Methods

Blood routine, blood coagulation function, liver and kidney function, and the infection index were examined before prostate biopsy. Patients took orally intestinal cleansing drugs before surgery and emptied stool in the morning of the day of surgery. A biplanar TRUS probe (Flex Focus 1202 rectal Ultrasound, BK, Denmark) was used with a silicone offset to lift the prostate anteriorly into an area that was accessible for perineal biopsy. The probe was attached to a brachytherapy stepping unit with a standard 0.5 cm brachytherapy template, and it was positioned over the perineum. Biopsy cores were obtained by a transperineal approach through the template grid using the Bard biopsy gun (Bard MCl 820) and an 18-gauge biopsy needle. The mean length of the biopsy cores was 22 mm. All of the biopsies were performed in the same operating room. The patients assumed the lithotomy position and were administered oxygen. According to the condition of prostate anatomy, the patients’ subjective and other factors, we chose the type of anesthesia. In the 4 subgroups of NBG, 90, 75, 79, 60 patients underwent prostate-coated local anesthesia along with PNB, respectively. For this anesthesia method, a total of 32 ml of 1% lidocaine was used, 10 ml of which was used in perineal area skin infiltration anesthesia, guided by intraoperative ultrasound, the scope of infiltration was 0.5 cm greater than the projection area; 12 ml lidocaine was rough injected to the perineum area in 1, 3, 5, 7, 9, and 11 points of prostate projection to get infiltration anesthesia of the apex of the prostate. The prostate blood vessel of prostatic neurovascular bundle could be clearly observed by TRUS. In the position of prostate blood vessel, 5 ml 1% lidocaine was injected using ANSll 0.5 mm ∗112mm spinal needle. Liquid anechoic area appearing between prostate and seminal vesicle was as the mark of anesthesia success. Same to the other side. The LAG underwent prostate-coated local anesthesia, the positions of the left and right nerve vessels were injected with normal saline 5 mL as control, and the remaining steps of the operation were the same as the NBG. A number of puncture needle and pain degree were recorded after puncture. Besides, vasovagal performance, such as dizziness, sweating, chest tightness, and shortness of breath, were closely observed. Visual analogue scale (VAS, 0, none; 10, intolerable pain)^[11]^ and visual numeric scale (VNS, 0, terrible; 4, perfect)^[12]^ were used to evaluate pain and quantify satisfaction.

### 2.3 Statistical analysis

SPSS 16 statistical software was used for data processing. Counting data were showed as  
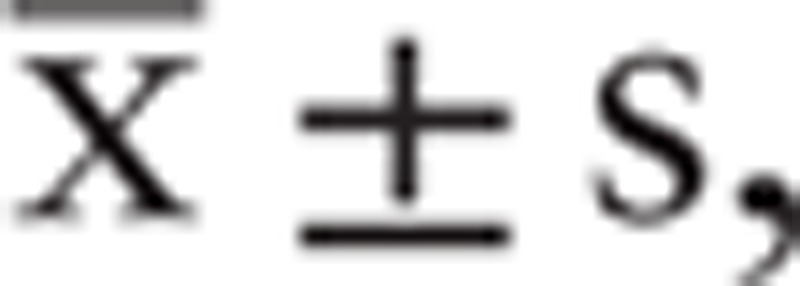
 whereas grade data were displayed as median (inter-quartile range). Nonparametric analysis included Mann-Whitney test and Kruskal-Wallis *H* test and were used to compare pain score. χ^2^ test was used to compare the rates of complication at intersubgroup or intrasubgroup level. The significance value was set at *P* < 0.05.

## 3 Results

Five hundred sixty-eight patients who underwent transperineal template-guided prostate biopsy were eligible for the study. The age and serum PSA level before biopsy had no significant differences at intergroup or intragroup level, as presented in Table 1.

**Table 1:**
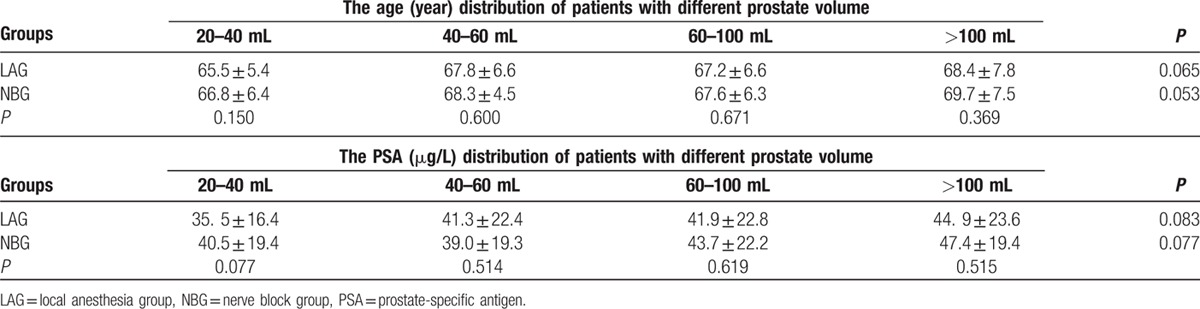
Distribution of age and PSA between the two groups  
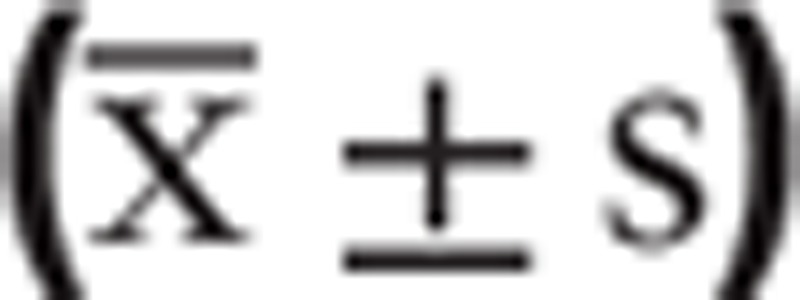
.

**Figure 1:**
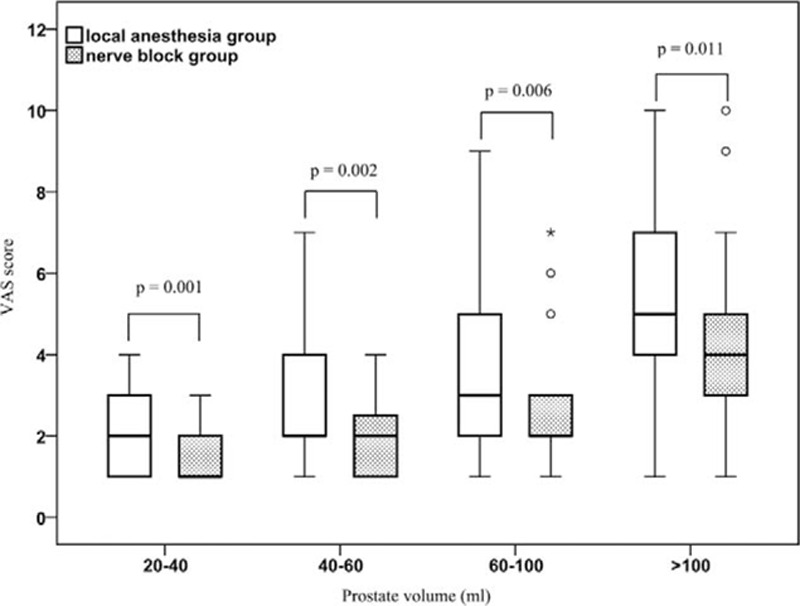
Box-plot of VAS scores in different anesthesia with different prostate volume. The difference of VAS scores between local anesthesia group and nerve anesthesia group were statistically significant, *P* values of each prostate volume subgroup were 0.001, 0.002, 0.006, and 0.011, respectively. The VAS scores also had significant difference between different prostate volume in local anesthesia group and nerve anesthesia group, respectively (both *P* < 0.001). VAS = visual analogue scale.

**Figure 2:**
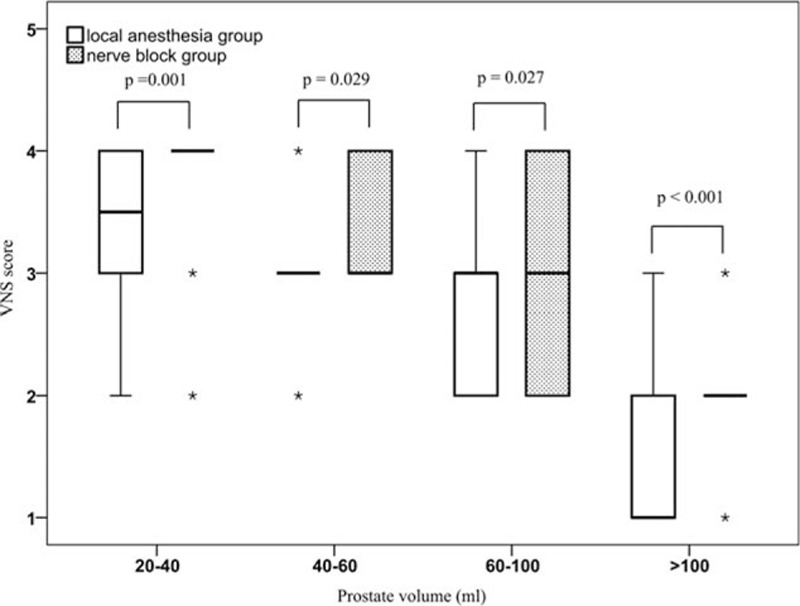
Box-plot of VNS scores in different anesthesia with different prostate volume. Compared with the local anesthesia group, the VNS scores were significantly higher in nerve anesthesia group in terms of prostate volume (all *P* < 0.05). In 2 groups, the VNS scores possess significant difference between different prostate volume (both *P* < 0.001). VNS = visual numeric scale.

Compared with the LAG, the VAS scores were significantly lower in the NBG in terms of prostate volume: 1 (1–2) versus 2 (1–3), 2 (1–3) versus 2 (2–4), 2 (2–3) versus 3 (2–5), 4 (3–5) versus 5 (4–7), respectively (*P* < 0.05; Table 2). The VNS scores were significantly higher in NBG with different prostate volume: 4 (3–4) versus 3.5 (3–4), 3 (3–4) versus 3 (3–3), 3 (2–4) versus 3 (2–3), 2 (2–2) versus 1 (1–2), respectively (*P* < 0.05; Table 3). The 4 subgroups of NBG were compared; it showed that the differences in VAS and VNS scores were statistically significant (*P* < 0.001, Figs. 1 and 2). The 4 subgroups of LAG also had significant differences (*P* < 0.001). In addition, we also observed that with the increase of prostate volume, the VAS score increased, whereas the VNS score decreased.

**Table 2:**
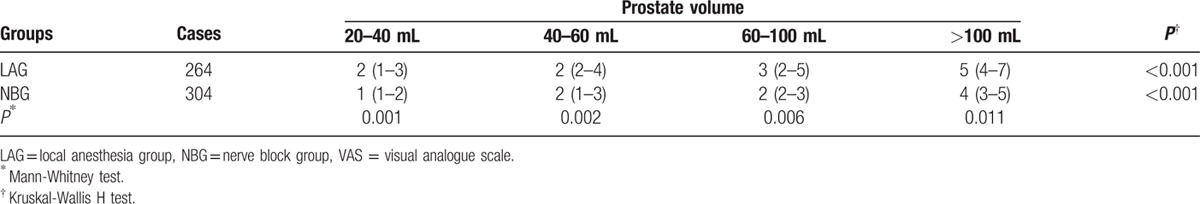
The VAS scores after prostate biopsy with different prostate volume.

**Table 3:**
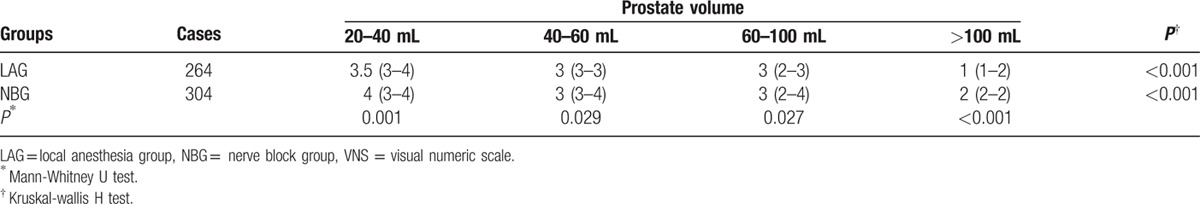
The VNS scores after prostate biopsy with different prostate volume.

Complications, including hematuria, infection, hemospermia, and urinary retention, had no statistically significant difference (*P* > 0.05) between NBG and LAG. But the hematuria, hemospermia and urinary retention at intersubgroups of LAG and NBG had statistically significant difference (*P* < 0.05) except infection (Table 4).

**Table 4:**
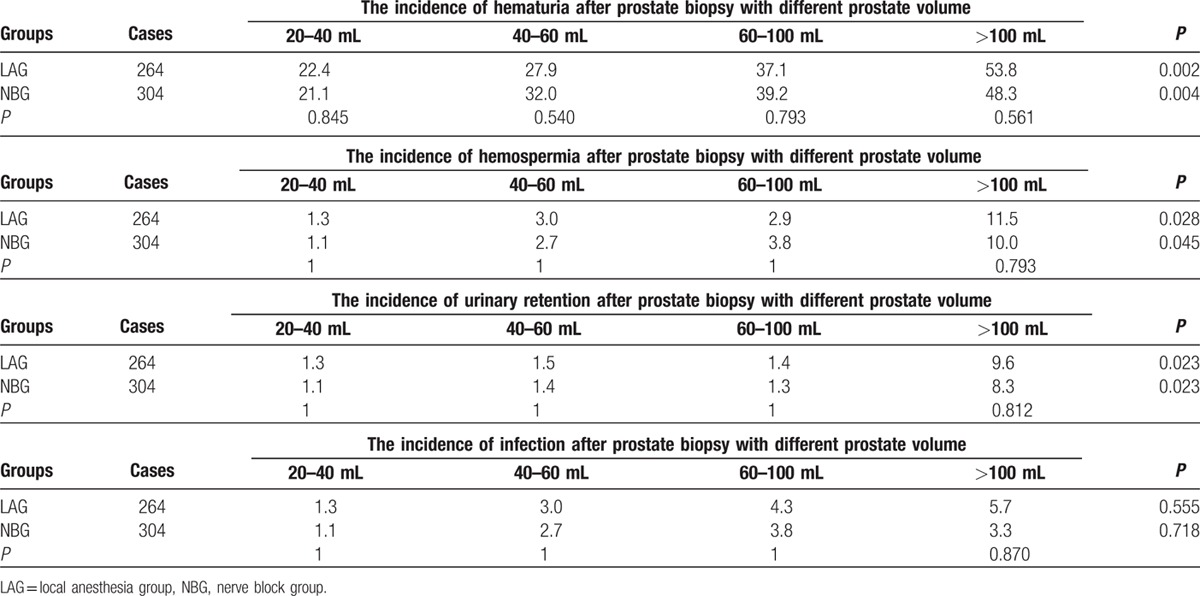
The complications of prostate biopsy.

## 4 Discussion

TRUS-guided transperineal prostate needle biopsy has become the most common method for the diagnosis of PCa. Currently, the main anesthesia used in prostate biopsy include PNB, perineal nerve block, rectum lidocaine surface anesthesia, and pelvic plexus nerve block anesthesia.^[13,14]^ PNB was an effective way of anesthesia for prostate puncture,^[15]^ which could directly infiltrate the neurovascular bundle.^[16]^ Conde et al^[17]^ found that bilateral PNB had better anesthetic effect than oral morphine. A meta-analysis conducted by Hergan,^[18]^ which included 14 studies, revealed that PNB was better than local anesthesia or placebo.

How to locate the periprostatic nerve is the key step of PNB. As is known, effective injection method must be based on accurate position of neurovascular bundle.^[19]^ In order to locate accurately, ultrasound was used to determine the depth and position of the needle. When injected anesthetics, we could see the liquid region of prostate and the corner of seminal vesicle which indicated the prostate was floating and the anesthetic injection site was accurate.^[13]^ Then, we performed transperineal template-guided prostate biopsy and used VAS and VNS to evaluate the anesthetic effect. Our study showed that the nerve block anesthesia was better than the traditional single capsule infiltration anesthesia for the patients with perineal prostate biopsy. But still, we also observed that in some patients who underwent PNB, it had poor effect. Apart from sensitivity to pain, most of these patients were with larger prostate volume. In view of this phenomenon, we had grouped the patients according to the volume of prostate to analyze the potential relationship between the effect of anesthesia and the volume of prostate. Among patients in NBG, we found that VAS and VNS scores were statistically significant in the pairwise comparison of the 4 subgroups (*P* < 0.05). Similarly, the 4 subgroups of LAG also have significant differences (*P* < 0.05), which suggested that the effect of prostatic anesthesia in patients with large prostate volume was poor.

For complications in NBG and LAG, difference had no statistical significance (*P* > 0.05). But the hematuria, hemospermia, and urinary retention at intersubgroups of LAG and NBG had statistically significant difference (*P* < 0.05) except infection. It manifested that puncture complications were closely related to the volume of the prostate rather than the anesthesia method. The difference in the effect of the prostatic anesthesia and complication might be due to incremental puncture number along with the increase of the prostate volume. As we know, with the increase of prostate volume, the positive rate turned low.^[20]^ As a consequence, more punctures were preferred to obtain more samples. In this case, patients might suffer from more pain, which is named “cumulative pain” by Kevar et al.^[21]^ In addition, unlike other solid tumors, the growth of the PCa was irregular.^[22]^ This made positioning of the prostate and seminal vesicle gland angle very difficult. Hence, how to locate the nerve vascular bundle accurately became the key step of prostate biopsy. At present, the method of locating the peripheral nerve of the prostate is to find the angle between the prostate and seminal vesicle. However, this method lacks accuracy. Facial nerve monitor in the Department of ENT (ear, nose and throat) might help to position the nerve vascular bundle. In the process of anesthesia, the monitor may also work. In future, we will try to use the waveform in the monitor to determine whether the position is accurate. In this way, we may easily target the peripheral nerve of the prostate. In addition, we used the same dose of anesthetics for any volume of prostate, and the anesthesia effect was poor for large prostate volume. Consequently, the relationship between the dose of anesthetics and the volume of the prostate will be studied to discuss the appropriate dose for different volume of prostate to achieve the optimal anesthetic effect.

Ultrasound-guided prostate peripheral nerve block anesthesia had great analgesic effect and equal safety, but for patients with a large prostate volume, the analgesic effect was inefficient. In this study, we did not consider the patient's level of education, local anesthetic sensitivity, physical health status, body mass index, time of anesthesia, and different operator on anesthetic effect. Hence, we need to continue to accumulate cases and study the PNB in depth for further optimization of the prostate. Large-scale prospective studies will be needed to further study this topic.

## References

[1] Siegel RL Miller KD Jemal A Cancer statistics, 2016. *CA Cancer J Clin* 2016; 66:7–30. 26742998 10.3322/caac.21332

[2] Chen W Zheng R Baade PD Cancer statistics in China, 2015. *CA Cancer J Clin* 2016; 66:115–132. 26808342 10.3322/caac.21338

[3] Heijnsdijk EA Wever EM Auvinen A Quality-of-life effects of prostate-specific antigen screening. *N Engl J Med* 2012; 367:595–605. 22894572 10.1056/NEJMoa1201637 PMC4982868

[4] Roobol MJ Kranse R Bangma CH Reply from authors re: Michael Baum. Screening for prostate cancer: can we learn from the mistakes of the breast screening experience? Eur Urol 2013;64:540-1: screening for prostate cancer: we have learned and are still learning. *Eur Urol* 2013; 64:541–543. 23816273 10.1016/j.eururo.2013.06.033

[5] Taira AV Merrick GS Galbreath RW Performance of transperineal template-guided mapping biopsy in detecting prostate cancer in the initial and repeat biopsy setting. *Prostate Cancer Prostatic Dis* 2010; 13:71–77. 19786982 10.1038/pcan.2009.42 PMC2834351

[6] Ding X Zhang L Zhou G The application of the template positioning transperineal prostate biopsy in the first prostate biopsy negative patients. *Chin J Urol* 2013; 34:298–300.

[7] Cerruto MA Vianello F D’Elia C Transrectal versus transperineal 14-core prostate biopsy in detection of prostate cancer: a comparative evaluation at the same institution. *Arch Ital Urol Androl* 2014; 86:284–287. 25641452 10.4081/aiua.2014.4.284

[8] Udeh EI Amu OC Nnabugwu II Transperineal versus transrectal prostate biopsy: our findings in a tertiary health institution. *Niger J Clin Pract* 2015; 18:110–114. 25511354 10.4103/1119-3077.146991

[9] Bingqian L Peihuan L Yudong W Intraprostatic local anesthesia with periprostatic nerve block for transrectal ultrasound guided prostate biopsy. *J Urol* 2009; 182:479–483. 19524987 10.1016/j.juro.2009.04.029

[10] Ukimura O Coleman JA de la Taille A Contemporary role of systematic prostate biopsies: indications, techniques, and implications for patient care. *Eur Urol* 2013; 63:214–230. 23021971 10.1016/j.eururo.2012.09.033

[11] Tufek I Akpinar H Atug F The impact of local anesthetic volume and concentration on pain during prostate biopsy: a prospective randomized trial. *J Endourol/Endourol Soc* 2012; 26:174–177. 10.1089/end.2011.0344 22092389

[12] Doganca T Savsin A Erdogan S Procedural sedation and analgesia as an adjunct to periprostatic nerve block for prostate biopsy: A prospective randomized trial. *J Clin Ultrasound* 2015; 43:288–294. 25155750 10.1002/jcu.22227

[13] Ding X Zhou G GX An analgesia study of periprostatic nerve block for transrectal ultrasound guided biopsy of the prostate. *Chin J Urol* 2014; 35:917–920.

[14] Sahin A Ceylan C Gazel E Three different anesthesia techniques for a comfortable prostate biopsy. *Urol Ann* 2015; 7:339–344. 26229322 10.4103/0974-7796.152014 PMC4518371

[15] Kumar A Griwan MS Singh SK Is periprostatic nerve block a gold standard in case of transrectal ultrasound-guided prostate biopsy? *Urol Ann* 2013; 5:152–156. 24049376 10.4103/0974-7796.115732 PMC3764894

[16] Obek C Ozkan B Tunc B Comparison of 3 different methods of anesthesia before transrectal prostate biopsy: a prospective randomized trial. *J Urol* 2004; 172:502–505. 15247714 10.1097/01.ju.0000131601.06286.26

[17] Conde Redondo C Alonso Fernandez D Robles Samaniego A TRUS-guided biopsy: comparison of two anesthetic methods. *Actas Urol Esp* 2006; 30:134–138. 16700202 10.1016/s0210-4806(06)73414-3

[18] Hergan L Kashefi C Parsons JK Local anesthetic reduces pain associated with transrectal ultrasound-guided prostate biopsy: a meta-analysis. *Urology* 2007; 69:520–525. 17382157 10.1016/j.urology.2006.12.005

[19] Maccagnano C Scattoni V Roscigno M Anaesthesia in transrectal prostate biopsy: which is the most effective technique? *Urol Int* 2011; 87:1–13. 21677420 10.1159/000327827

[20] Leibovici D Shilo Y Raz O Is the diagnostic yield of prostate needle biopsies affected by prostate volume? *Urol Oncol* 2013; 31:1003–1005. 21924650 10.1016/j.urolonc.2011.08.008

[21] Kaver I Mabjeesh NJ Matzkin H Randomized prospective study of periprostatic local anesthesia during transrectal ultrasound-guided prostate biopsy. *Urology* 2002; 59:405–408. 11880081 10.1016/s0090-4295(01)01538-2

[22] Konyalioglu E Tarhan H Cakmak O Prostate cancer volume estimations based on transrectal ultrasonography-guided biopsy in order to predict clinically significant prostate cancer. *Int Braz J Urol* 2015; 41:442–448. 26200537 10.1590/S1677-5538.IBJU.2014.0251 PMC4752136

